# Sex Differences in the Association Between Drinking Motives, Protective Behavioral Strategies, and Alcohol Outcomes in a Hazardous Drinking College Sample

**DOI:** 10.1080/10826084.2026.2682302

**Published:** 2026-06-15

**Authors:** Jillian E. Hardee, Katie J. Paige, Zachary D. Ekves, Omid Kardan, Katherine L. McCurry, Meghan E. Martz, Lora M. Cope

**Affiliations:** a Department of Psychiatry, University of Michigan, Ann Arbor, Michigan; b Addiction Center, University of Michigan, Ann Arbor, MI

**Keywords:** Alcohol, sex differences, drinking motives, protective behavioral strategies

## Abstract

**Background:**

Drinking motives and protective behavioral strategies have been shown to impact the frequency and quantity of alcohol consumption in young adults, including college students who are at high risk for hazardous drinking. However, how men and women employ different protective behavioral strategies with respect to their drinking motives, and whether these impact drinking patterns differentially, are currently understudied.

**Methods:**

Data from 109 college students with hazardous drinking patterns were analyzed to examine effects of drinking motives, protective behavioral strategies, sex, and their interactions on four different drinking outcomes.

**Results:**

Men reported significantly higher values for drinks, drinking days, drinks per drinking day, and enhancement-related drinking motives. Women had significantly higher values for harm reduction-related protective behavioral strategies. Linear regression analyses demonstrated significant 2- or 3-way interactions in three of the four alcohol outcomes we examined. In addition, all but one of these interactions included sex.

**Conclusion:**

Our findings highlight the importance of examining sex differences in alcohol-related behaviors, cognitions, and outcomes among college students and demonstrate the nuance and complexity of such alcohol-related factors. As such, findings from the current study may inform the development of more targeted alcohol use prevention and interventions efforts on college campuses.

## Introduction

Young adult men typically consume alcohol more frequently and in greater quantities per occasion and are more likely to engage in binge drinking compared to young adult women (Patrick et al., 2024; Slutske, 2005; White, 2020; White & Hingson, 2013), though the gap is narrowing (White, 2020). Sex differences in drinking behaviors can be influenced by several different factors, such as protective behavioral strategies and drinking motives.

Drinking motives, such as drinking to cope or fit in socially, are a particularly strong determinant of drinking behaviors (Cooper, 1994; Cox & Klinger, 1988). These motives are a cognitive explanation for drinking behavior, the consequences of which can be categorized along two axes: the type of reinforcement (positive vs. negative) and the source of motivation (internal vs. external) (Cox & Klinger, 1988). Motives that correspond to positively reinforcing consequences can be internally (e.g., enhancing one’s mood; “enhancement”) and externally (e.g., improving social interactions; “social”) driven. Similarly, motives that correspond to negatively reinforcing consequences can be internally (e.g., reducing one’s negative mood; “coping”) and externally (e.g., avoiding negative social interactions or avoiding peer disapproval; “conformity”) driven. Multivariate analyses from numerous studies indicate that internal motives (enhancement, coping) most reliably predict alcohol consumption while motives that correspond to negatively reinforcing consequences (coping, conformity) are most directly linked to alcohol-related consequences (Ham & Hope, 2003; Kuntsche et al., 2005).

In general, men are more likely to cite motives related to positive reinforcement while women are more likely to cite motives related to negative reinforcement. For example, men often cite drinking to enhance positive moods and experiences as their primary motives for drinking—using alcohol to boost their confidence in social situations—or to cope with external stressors, such as social pressure (e.g., Erol & Karpyak, 2015). Women are more likely to drink to cope with internal stressors (e.g., depression, anxiety), using alcohol to manage feelings of sadness and loneliness or to alleviate anxiety (e.g., Peltier et al., 2019, but also see Foster et al., 2014). These differences manifest in distinct behavioral patterns: men typically drink heavily but less frequently while women may drink more frequently at lower amounts, often integrating drinking into daily routines to cope (Kenney et al., 2015; Wilsnack et al., 2018). Notably, drinking to cope with internal and external stressors are both associated with alcohol-related consequences (Bresin & Mekawi, 2021; Wardell et al., 2020), emphasizing the predictive importance of drinking motives for both consumption and alcohol-related consequences (Merrill et al., 2014).

Although strong drinking motives can drive alcohol use and related harms, the use of protective behavioral strategies, which are deliberate actions that individuals take to reduce the amount of consumption and/or negative consequences associated with drinking, may counteract this influence. Protective behavioral strategies encompass a range of behaviors, such as setting drinking limits, alternating between alcoholic and nonalcoholic beverages, and avoiding drinking games (Martens et al., 2005). While the literature strongly supports the efficacy of protective behavioral strategies in reducing alcohol use and consequences, findings are less consistent across subtypes of strategies (Cox et al., 2024). For example, manner of drinking (e.g., avoid mixing different types of alcohol) and limiting/stopping (e.g., leave the bar/party at a predetermined time) strategies are more effective in reducing heavy drinking than are serious harm reduction strategies (Cox et al., 2024; Peterson et al., 2021). For alcohol-related consequences, serious harm reduction and manner of drinking often predict fewer consequences, but some studies report unexpected positive correlations (see Cox et al., 2024 for a review). Furthermore, the majority of studies looking at limiting/stopping strategies find no association with alcohol-related consequences. These varied results, along with the fact that far fewer studies have examined consumption (as opposed to consequences) (Cox et al., 2024), highlight the need to examine multiple alcohol outcomes (e.g., quantity, consequences).

There is clear evidence of sex differences in the use and effectiveness of protective behavioral strategies; women are more likely to use protective behavioral strategies compared to men (Labrie et al., 2011; Pearson, 2013; Tabernero et al., 2019). Several studies have examined whether sex moderates the relationship between protective behavioral strategies and alcohol-related outcomes, but findings are inconsistent. Some studies report protective behavioral strategies are more strongly associated with reduced alcohol-related outcomes in women compared to men (Clarke et al., 2016; Montes et al., 2019); however, others find no evidence of sex differences after accounting for alcohol use levels (Li et al., 2022). Notably, most studies have relied on total protective behavioral strategies scores; yet, Miller et al. (2019) examined PBSS subtypes (e.g., stopping/limiting, manner of drinking, serious harm reduction) and found that sex differences were evident for some subtype–outcomes (e.g., manner of drinking and consequences) but not others, underscoring the possibility that sex moderation varies by PBS subtype and by alcohol-related outcome. We hypothesize the moderating effect of sex in the association between the use of protective behavioral strategies and alcohol-related outcomes may depend on the subtype of protective behavioral strategy that is employed.

Two of the major drivers that impact how much an individual drinks and what associated consequences they experience are motives and protective strategies. Prior research has examined combinations of protective behavioral strategies, drinking motives, and drinking outcomes only in isolation or in pairwise combinations (Labrie et al., 2011; Linden et al., 2014; 2014; Looby et al., 2019), potentially obscuring important distinctions in how motives and strategies manifest across patterns of drinking behavior and alcohol-related consequences. Thus, gaps remain in our understanding of how these constructs interact, particularly with respect to sex differences. To our knowledge, only one study has directly tested whether protective behavioral strategies can moderate the association between drinking motives and drinking outcomes in college students (Patrick et al., 2011), but no one has further explored whether these interactions differ meaningfully between men and women.

Given these gaps, our study aimed to replicate and extend previous work by examining whether the use of protective behavioral strategies and drinking motives differs by sex (Objective 1), and whether interactions between these factors help explain persistent differences in alcohol-related outcomes between men and women (Objective 2). We also examined four different drinking-related outcomes (drinks, drinking days, drinks per drinking day, and alcohol-related consequences). This approach allowed us to systematically examine how drinking motives, protective behavioral strategies, and their interactions are related to drinking patterns in college-aged men and women. Based on the prior literature, we hypothesized that men would report higher alcohol use, while women would use protective behavioral strategies more frequently. We further expected women to cite coping and conformity motives and men to cite enhancement and social motives as primary drivers of their drinking behaviors. Our main aim was to address ongoing questions about how sex may influence the connections between drinking motives, protective behavioral strategies, and drinking outcomes. Because previous studies have not examined how sex, motives, and protective strategies interact, we did not make hypotheses about any interactions. Given the high prevalence of other substance use in young adult populations (i.e., cannabis, nicotine, and other drugs), we controlled for other substance use in our primary analysis.

## Materials and methods

### Participants

Participants were 109 college students (mean age = 19.4, *SD* = 0.8; 51.4% women) from a single, large, midwest university in the United States, recruited for a study examining sex differences in the effects of a brief intervention on alcohol use and brain function. The data used in the present study are from the baseline session, which occurred prior to any intervention (control *n* = 69; 35 women; intervention *n* = 40; 21 women; see Supplemental Material for more details). Participants provided written informed consent, and the study was approved by the university Institutional Review Board. In this study, “sex” refers to “sex assigned at birth.” Demographic information is displayed in Table 1.

To qualify for the study, individuals in both groups had to: 1) screen positive for hazardous drinking *via* the Alcohol Use Disorders Identification Test–Consumption (AUDIT-C; Bush et al., 1998; see below for more details), 2) be enrolled full-time in college, and 3) be 18–20 years old. Exclusionary criteria were: 1) neurological, acute, uncorrected, or chronic medical illness, 2) current treatment for or diagnosis of any of the following: schizophrenia, post-traumatic stress disorder, bipolar disorder, major depressive disorder, an eating disorder, obsessive compulsive disorder, panic disorder, social phobia, agoraphobia, or generalized anxiety disorder), 3) magnetic resonance imaging contraindications such as metal implants, medical devices, pregnancy, or claustrophobia, and 4) left-handedness or ambidexterity. The latter two criteria are due to the broader study protocol also including neuroimaging.

### Questionnaires

#### Alcohol

The AUDIT-C was collected at the screening phase to determine eligibility and assessed drinking behavior over the previous 90 days. The AUDIT-C consists of the first three items of the full-scale AUDIT and assesses frequency of drinking, typical number of drinks consumed on a drinking day, and frequency of heavy drinking (Bush et al., 1998). Responses to each question are scored 0–4, yielding a maximum score of 12. To screen positive for hazardous drinking, women had to score ≥4 and men had to score ≥5; these cutoff scores were selected as they are optimal for classifying the highest proportion of individuals as having hazardous-drinking habits (Khadjesari et al., 2017) and because of research illustrating that women have higher blood alcohol levels after drinking the same amount of alcohol as men, even when controlling for body weight, suggesting higher risk at lower levels of alcohol use (Wechsler et al., 1995).

The Timeline Followback (TLFB; Sobell & Sobell, 1992) was used to obtain a retrospective report on daily drinking over the 30 days prior to the baseline session. Participants used a calendar to provide a record of their daily drinking (1 drink = 12 oz beer, 5 oz glass of wine, or 1.5 oz shot of liquor). From this the number of drinks consumed, number of drinking days, and number of drinks per drinking day in the past 30 days were calculated.

The Rutgers Alcohol Problem Index (RAPI) was used to assess alcohol-related consequences (White & Labouvie, 1989). Participants were asked the number of times they experienced each of 18 negative consequences (e.g., missing class, going to class drunk) because of drinking in the 30 days prior to their baseline session. Responses to each question are scored 0–4, yielding a maximum score of 72. In the present study, Cronbach’s alpha was 0.766.

The Drinking Motives Questionnaire-Revised (DMQ-R; Cooper, 1994) was used to assess reasons for drinking alcohol. Each of the 20 items is rated on a 5-point scale indicating how frequently the given reason serves as motivation to drink alcohol (1 = *almost never/never;* 5 = *almost always/always*). There are four subscales, each with five items: Social (e.g., “To celebrate a special occasion with friends”), Coping (e.g., “To forget your worries”), Enhancement (e.g., “Because you like the feeling”), and Conformity (e.g., “To fit in with a group you like”). Cronbach’s alphas were: 0.757 (Social), 0.798 (Coping), 0.760 (Enhancement), and 0.707 (Conformity).

The Protective Behavioral Strategies Survey (PBSS; Martens et al., 2005) was used to assess strategies to limit alcohol use and consequences. The 15 items, each rated on a 6-point scale (1 = *never*; 5 = *always*; scored in such a way that higher scores indicated use of more protective behavioral strategies), index 3 subscales: Limiting/Stopping Drinking (e.g., “Drink water while drinking alcohol”; 7 items), Manner of Drinking (e.g., “Avoid drinking games”; 5 items), and Harm Reduction (e.g., “Know where your drink has been at all times”; 3 items). In the present study, Cronbach’s alphas were: 0.732 (Limiting/Stopping Drinking), 0.523 (Manner of Drinking), and 0.774 (Harm Reduction). For two of the subscales, PBSS Manner of Drinking and PBSS Harm Reduction, there were items that had extremely low item-total correlations and were therefore removed. Those items were: PBSS Item 12 from Manner of Drinking (“Avoid trying to ‘keep up’ or out-drink others”; *r* = 0.07) and PBSS Item 13 from Harm Reduction (“Use a designated driver”; *r* = 0.10). The Cronbach’s alphas reported above as well as all other analyses use these re-calculated subscales with those items removed. Even after removing Item 12 from Manner of Drinking, Cronbach’s alpha remained problematically low (i.e., 0.523), so Manner of Drinking was not included in the models described below.

#### Other substance use

The TLFB was also used to obtain a retrospective report of cannabis use over the 30 days prior to the baseline session (Sobell & Sobell, 1992). Participants used a calendar to provide a record of their daily cannabis use, measured in grams, over the past 30 days (1 joint = 0.5 grams).

The Drug History Questionnaire (DHQ) was used to quantify illicit drug use (i.e., other than alcohol and cannabis; Sobell et al., 1995). The number of “yes” responses a participant gave when asked if they had ever used a drug from nine different drug classes was summed, giving a value of 0 to 9 for each participant, indexing the number of drug classes used in their lifetime. The Adult Self-Report (Achenbach & Rescorla, 2003) was used to determine if a participant had any nicotine use in the 6 months prior to the baseline session.

Following Hardee et al. (2024) and Weigard et al. (2021), the three individual non-alcohol substance use variables described above (cannabis quantity over the 30 days prior to the baseline session, number of illicit drugs out of 9 used in the lifetime, any nicotine use in the 6 months prior to the baseline session) were used to create a composite measure indexing an individual’s relative amount of substance use other than alcohol compared to other individuals in the sample. To do this, we converted the three substance use measures to Z-scores and then averaged across them to obtain one substance use composite.

#### Analyses

As part of descriptive analyses, Welch’s *t*-tests or chi-square tests were run in SPSS 28 to assess sex differences in age, race/ethnicity, substance use composite, and alcohol outcomes. In addition, zero-order correlations indexed the strength and direction of the relationship among substance use, DMQ, and PBSS variables. Finally, although we note that the data analyzed here were collected pre-intervention, because assignment to the intervention or control group was not random (see Supplemental Material), we additionally examined potential differences between groups on sex, DMQ subscales, and PBSS subscales.

For Objective 1, Welch’s *t*-tests or chi-square tests were run to assess sex differences in DMQ and PBSS subscales.

For Objective 2, regression models were run in base R (R Core Team, 2024). To alleviate concerns related to overfitting and the number of possible comparisons, we conducted repeated *k*-fold cross validation (using the cvms R package; Olsen & Zachariae, 2024) to identify best performing models. Here we split the data into *k* = 10 folds/subsets (based on suggestions from Molinaro et al., 2005) and computed model statistics on each fold. This process was repeated a total of 10 times, resulting in 100 total folds of the data. Then we averaged across all folds to assess average model fit and selected the best performing model as indicated by lowest Akaike Information Criterion (AIC) score, which considers the tradeoff between increasing model fit and complexity.

Because a full exploration of all possible combinations of the DMQ and PBSS subscales—along with sex—would be computationally massive, we limited the analysis to include three predictors of interest in a single model according to the following general scheme: alcohol outcome ~ sex + DMQ subscale + PBSS subscale. The four alcohol outcomes (drinks, drinking days, drinks per drinking day, and alcohol-related consequences) were tested in separate models. Each best-fitting model could contain only one DMQ subscale and PBSS subscale, though sex was always a potentially available predictor (i.e., DMQ Social could not exist in the same model as DMQ Conformity). We further allowed for all possible interactions (of up to 3 terms) in each model. Prior to being entered into the model, all predictors were scaled and centered unless otherwise noted.

In addition to sex, DMQ subscale, and PBSS subscale, all models included group (intervention group, control group) and other substance use; both group and other substance use were required to be in the best-fitting model. The rationale for including group as a covariate, despite using only data from the baseline session in the present analyses, is that participants were not randomized to the intervention or control group (see Supplemental Material for details). Other substance use was included because of the high prevalence of use of other substances in young adult populations, where individuals who engage in hazardous drinking are more likely to report higher rates of other substances (e.g., Patrick et al., 2019). For models with alcohol-related consequences (RAPI) as the dependent variable, we also included drinks as a control so that the observed effects would be specific to consequences, holding quantity of drinks constant. For completeness, we also ran models without covariates (i.e., sex, DMQ, and PBSS predicting alcohol outcome but without group and other substance use), in which no terms were required to be in the best-fitting model. Results were substantively the same as when group and other substance use were included (see Supplemental Material).

After we identified the best performing model for each outcome using repeated 10-fold cross-validation, we reran the model using the full set of data. We used Breusch-Pagan tests (lmtest package; Zeileis & Hothorn, 2002) to assess heteroskedasticity of error. In cases where significant levels were shown, we used robust model estimation (type HC3) as implemented in the sandwich package (Zeileis, 2004; Zeileis et al., 2020). Based on suggestions that count data regularly violate standard assumptions in linear modeling (Tan et al., 2024), we used zero-inflated negative binomial models for drinks and drinking days. Alcohol-related consequences and drinks per drinking day were fit using linear models.

## Results

Descriptives: Sex was not significantly associated with age (*t*(107) = 0.83, *p* = 0.205) or race/ethnicity (*X*^2^(6) = 9.35, *p* = 0.155), but sex did have a significant effect on substance use composite (*t*(107) = −3.03, *p* = 0.003), where men had a higher mean than women (Table 1). Men also had significantly higher values of drinks, drinking days, and drinks per drinking day. Men also had higher AUDIT-C scores (measured at screening; *t*(107) = −3.21, *p* < 0.001; Table 1). Zero-order correlations among study variables can be found in Table 2. Regarding differences by intervention/control group on the DMQ subscales, PBSS subscales, and sex, Welch’s *t*-tests or chi-square tests indicated that the intervention and control groups only differed significantly on PBSS Limiting/Stopping (intervention > control; *t*(97.3) = −3.05, *p* = 0.002).

Objective 1: Men had significantly higher values of DMQ Enhancement. Women had significantly higher values for PBSS Harm Reduction. See Table 3.

### Regression models

Results corresponding to Objective 2 are below (with other substance use and group as covariates). See Table 4 for model statistics.

### Drinks

**Figure 1.**
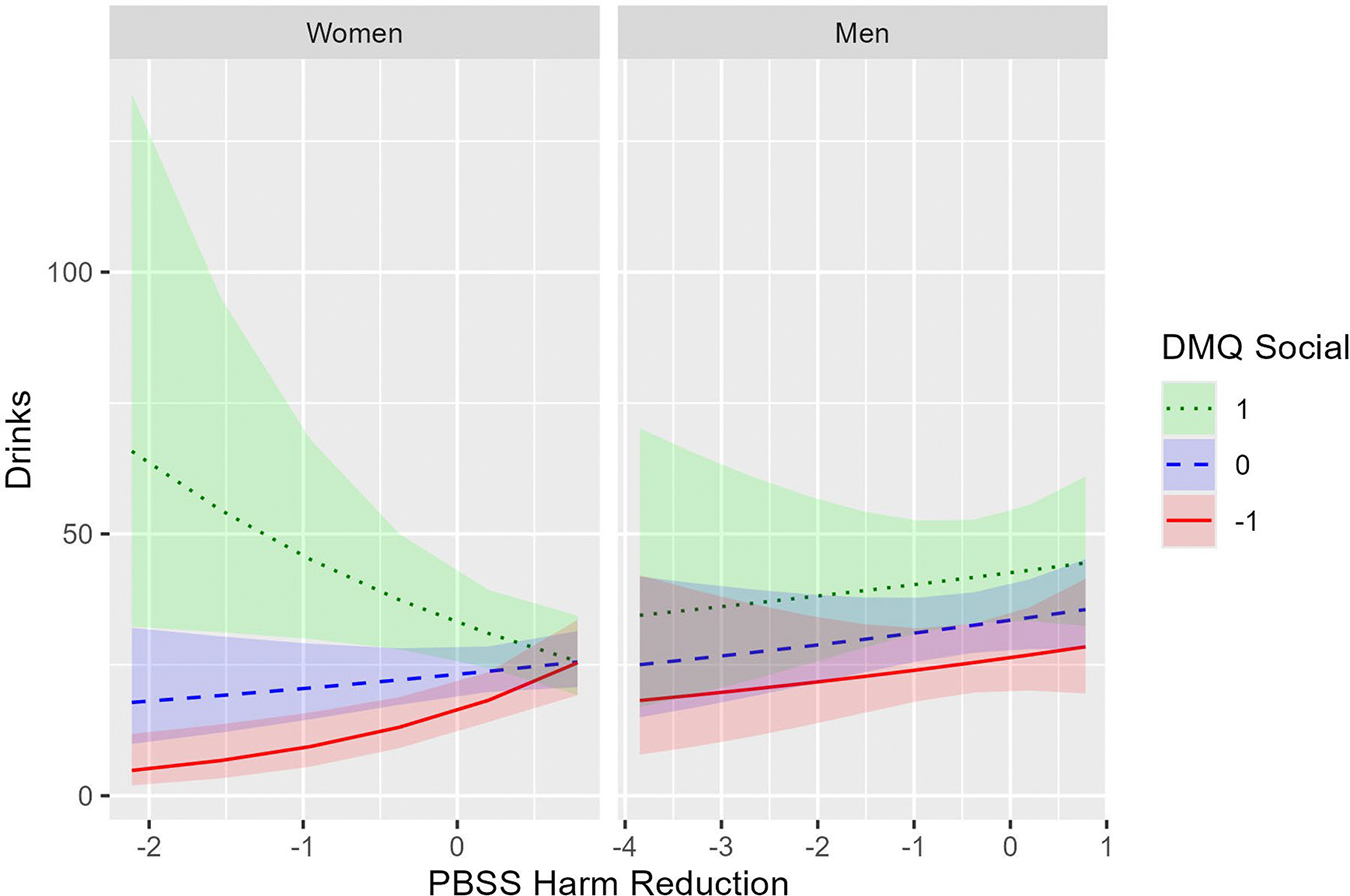
Interaction of DMQ social x PBSS harm reduction x sex on drinks. In women (left panel), at low levels of DMQ social (red line), there was a significantly positive relationship between PBSS harm reduction and drinks. At high DMQ social (green line), there was a significantly negative relationship between PBSS harm reduction and drinks. At average DMQ social levels (blue line), there was no significant relationship between PBSS harm reduction and drinks. No significant relationship was shown for men at low, average, or high DMQ social scores (right panel). To account for differences in scale, predictors were mean centered and standardized. Note that the x-axis is different for women and men due to a difference in the range of observed values.

The best-fitting model predicting drinks (*R*^2^_adj_ = 0.992) included a significant 3-way interaction between DMQ Social x PBSS Harm Reduction x sex (*p* = 0.002). To better understand the nature of the 3-way interaction effect, we analyzed simple slopes. At low DMQ Social (1 SD below the mean), women had a significantly positive relationship between PBSS Harm Reduction and drinks (*β* = 0.574, *p* = 0.001). At high DMQ Social (1 SD above the mean), women had a significantly negative relationship between PBSS Harm Reduction and drinks (*β* = −0.325, *p* = 0.028). At average DMQ Social levels (at group mean), women had no significant relationship between PBSS Harm Reduction and drinks (*β* = 0.124, *p* = 0.289). No significant relationship was shown for men at low (*β* = 0.097, *p* = 0.416), average (*β* = 0.076, *p* = 0.286), or high DMQ Social scores (*β* = 0.055, *p* = 0.572). See Figure 1. There was also a significant 2-way interaction between DMQ Social x PBSS Harm Reduction (*p* < 0.001) and significant main effects of sex (*p* < 0.001) and DMQ Social (*p* < 0.001). Due to the presence of the 3-way interaction, these lower order effects were not further interpreted.

### Drinking days

The best-fitting model predicting drinking days (*R*^2^_adj_ = 0.624) included a significant main effect of DMQ Enhancement (*p* = 0.049), such that higher levels of DMQ Enhancement were associated with more drinking days. There was also a significant main effect of other substance use (*p* = 0.019), such that greater use of other substances was associated with more drinking days.

### Drinks per drinking day

**Figure 2.**
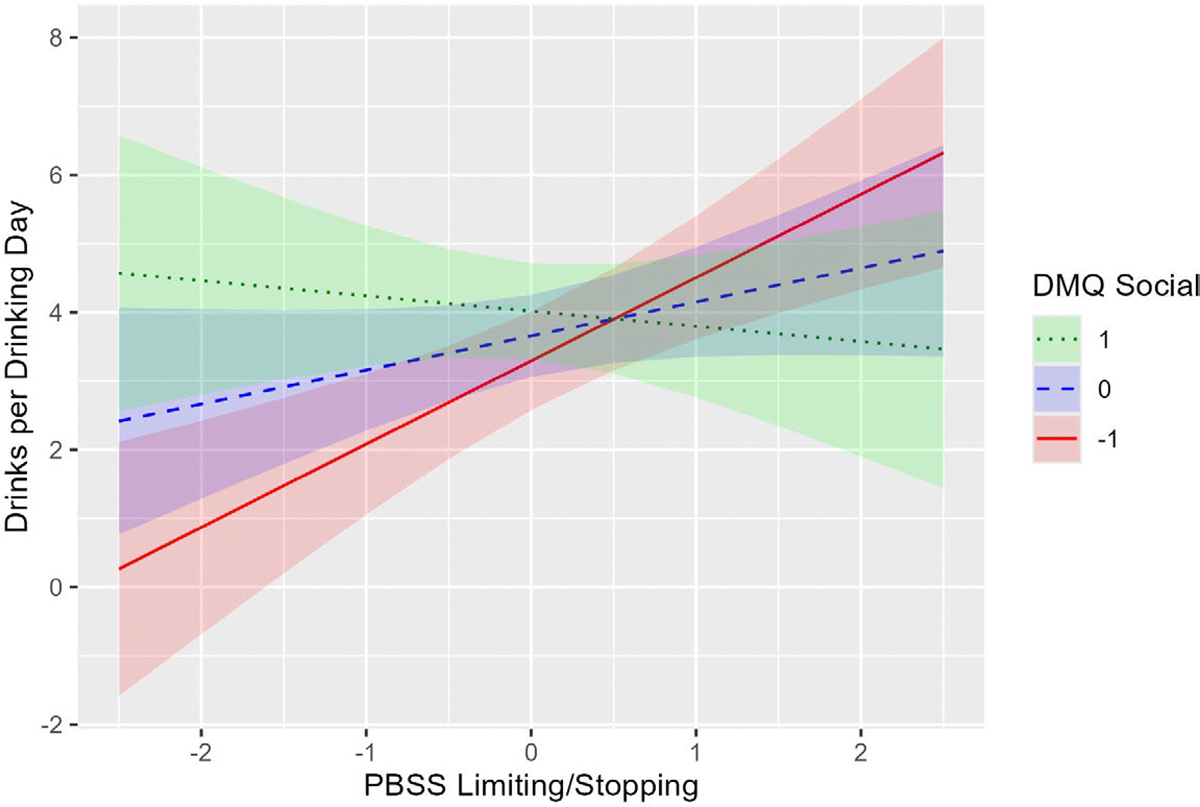
Interaction of DMQ social x PBSS limiting/stopping on drinks per drinking day. At low levels of DMQ social (red line), higher PBSS limiting/stopping scores (i.e., higher levels of protective strategies) were significantly associated with more drinks per drinking day. In contrast, at high levels of DMQ social (green line), higher PBSS limiting/stopping scores were significantly associated with fewer drinks per drinking day. To account for differences in scale, predictors were mean centered and standardized.

**Figure 3.**
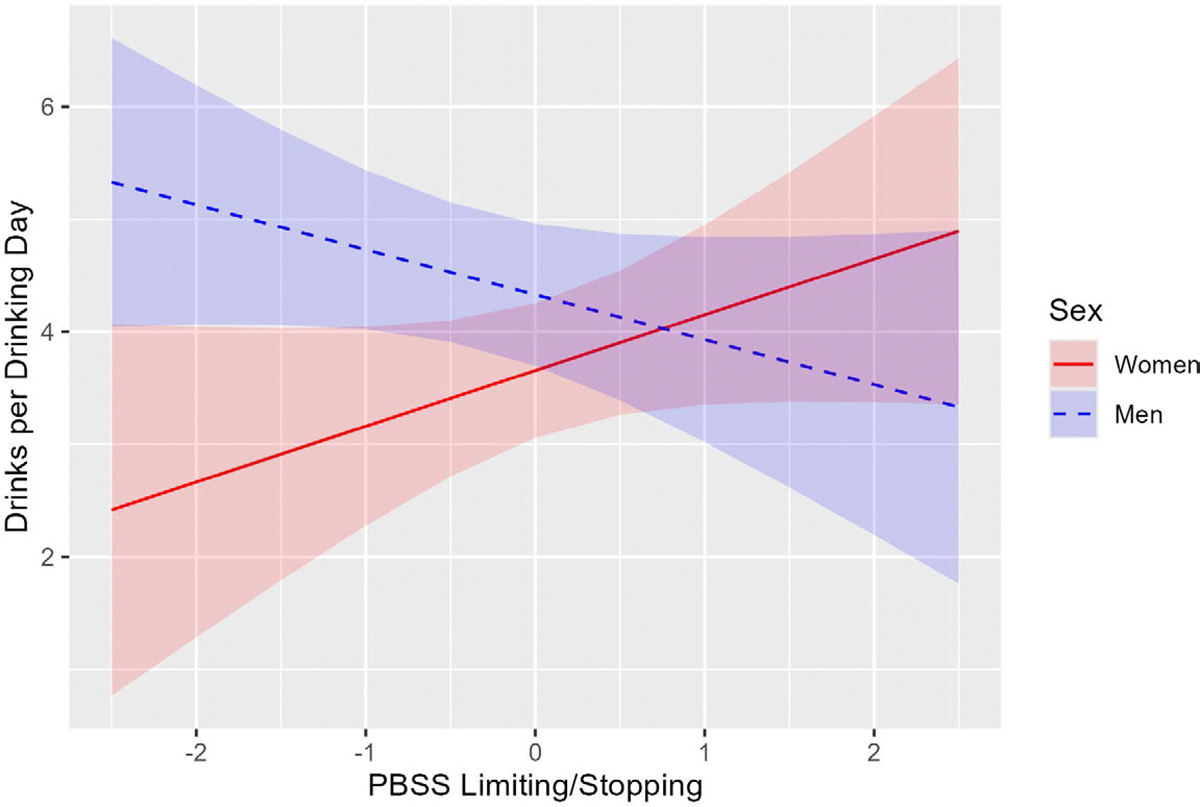
Interaction of sex x PBSS limiting/stopping on drinks per drinking day. In men (blue line), higher PBSS limiting scores were associated with fewer drinks per drinking day (trend-level). In women (red line), PBSS limiting/stopping scores were associated with more drinks per drinking day (trend-level). To account for differences in scale, predictors were mean centered and standardized.

The best-fitting model predicting drinks per drinking day (*R*^2^_adj_ = 0.226) included two significant 2-way interaction terms: DMQ Social x PBSS Limiting/Stopping (*p* < 0.001) and PBSS Limiting/Stopping x sex (*p* = 0.023). There were trend level main effects for sex (*p* = 0.087) and DMQ Social (*p* = 0.062). At low levels of DMQ Social (−1 SD below the mean), higher PBSS Limiting/Stopping scores (i.e., higher levels of protective strategies) were significantly associated with more drinks per drinking day (*β* = 0.765, *p* = 0.005). In contrast, at high levels of DMQ Social (+1 SD above the mean), higher PBSS Limiting/Stopping scores were significantly associated with fewer drinks per drinking day (*β* = −0.669, *p* = 0.022). At average levels of DMQ Social, there was no significant association between PBSS Limiting/Stopping and drinks per drinking day (*β* = 0.048, *p* = 0.812). See Figure 2. For the PBSS Limiting/Stopping x sex interaction, in men, higher PBSS Limiting scores were associated with fewer drinks per drinking days, although this effect was not significant (*β* = −0.399, *p* = 0.127). In women, however, higher PBSS Limiting/Stopping scores were associated with more drinks per drinking day at trend-level (*β* = 0.495, *p* = 0.100). See Figure 3.

### Alcohol-related consequences

**Figure 4.**
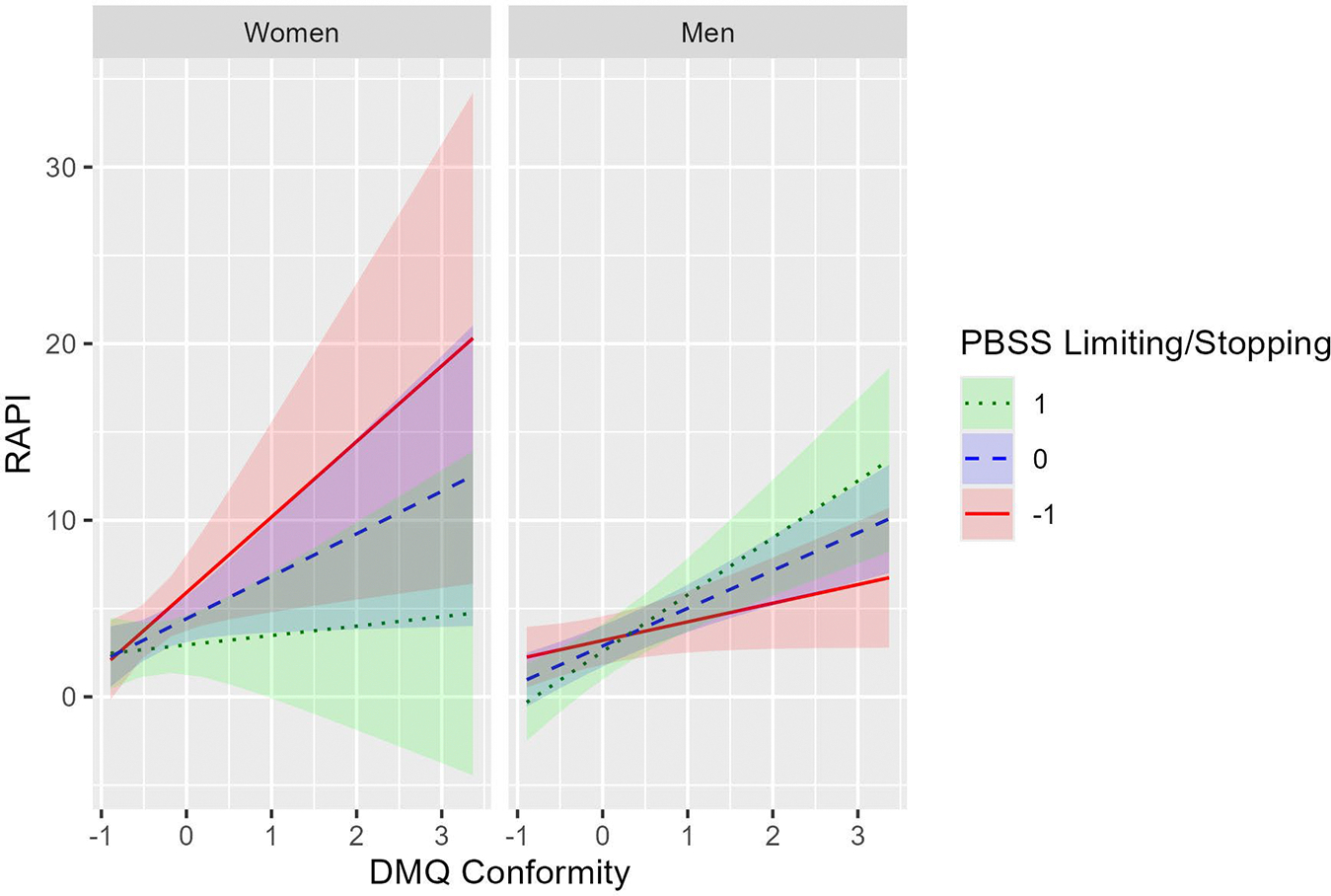
Interaction of PBSS limiting/stopping x DMQ conformity x sex on alcohol-related consequences (RAPI). In women (left panel), at low (red line) and average (blue line) levels of PBSS limiting/stopping, higher DMQ conformity scores were associated with higher RAPI scores. This direction was consistent with high PBSS limiting/stopping scores (green line), though not significant. In men (right panel), higher DMQ conformity scores were associated with higher RAPI scores at all levels of PBSS limiting/stopping. These effects were significant at average (blue line) and high (green line) PBSS limiting/stopping scores but only at trend-level at low scores (red line). To account for differences in scale, predictors were mean centered and standardized.

The best-fitting model predicting RAPI total scores (also controlling for total drinks; *R*^2^_adj_ = 0.351) included a significant 3-way interaction between DMQ Conformity x PBSS Limiting/Stopping x sex (*p* < 0.001) (Figure 4). There was also a 2-way interaction between DMQ Conformity x PBSS Limiting/Stopping (*p* = 0.005) and significant main effects of DMQ Conformity (*p* < 0.001), PBSS Limiting/Stopping (*p* = 0.010), sex (*p* = 0.045), group (*p* = 0.001), drinks (*p* = 0.040), and substance use control (*p* = 0.014). Results without controlling for drinks were substantively the same.

For women, at low (−1 SD below mean) and average (at group mean) levels of PBSS Limiting/Stopping, higher DMQ Conformity scores were associated with higher RAPI scores (*β* = 4.278, *p* < 0.001; *β* = 2.405, *p* < 0.001). High PBSS Limiting/Stopping scores (+1 above mean) showed a consistent direction of effect, but it was not significant (*β* = 0.532, *p* = 0.468).

For men, higher DMQ Conformity was associated with higher RAPI scores at all levels of PBSS Limiting/Stopping. These effects were significant at average and high PBSS Limiting/stopping scores (*β* = 2.139, *p* < 0.001; *β* = 3.222, *p* < 0.001); however, they were only trend-level at low scores (*β* = 1.055, *p* = 0.076). All values reported for alcohol-related consequences were corrected using robust estimation to account for heteroskedastic error.

## Discussion

This study examined how drinking motives and protective behavioral strategies interact to predict drinking outcomes in college students who engage in hazardous drinking, with a focus on sex differences. We found that sex differences emerged in the use of certain protective behavioral strategies and drinking motives. Consistent with our hypothesis and prior work (Ham et al., 2009; Kuntsche et al., 2006; Wild et al., 2001), men reported greater use of enhancement motives compared to women. Our finding that women more frequently employed harm reduction strategies replicates and extends prior work, which focused on overall protective strategies use rather than comparing types of strategies (Benton et al., 2004; Labrie et al., 2011) and general college samples rather than college students with hazardous drinking as done here (Delva et al., 2004; Schwebel et al., 2022; Sutfin et al., 2009). Together, these findings are similar to prior research showing sex-based differences in both drinking intentions and risk mitigation behaviors. For regression models, results revealed that alcohol outcomes were shaped by complex interactions between drinking motives, protective behavioral strategies, and sex, with 3-way and 2-way interactions indicating that the effectiveness and direction of protective behavioral strategies depended on individuals’ motives for drinking and their sex.

### Drinks: Association of harm reduction strategies and drinks consumed in women depends on their level of social motives

For drinks, we found a significant 3-way interaction among social motives, harm reduction strategies, and sex in predicting the number of drinks consumed. For women, the association between harm reduction strategies and drinks varied based on their level of social motives for alcohol use. Specifically, in women who reported low levels of social drinking motives, greater use of harm reduction strategies was associated with more drinks consumed. Conversely, among women with high levels of social drinking motives, greater use of harm reduction strategies was associated with fewer drinks consumed. There were no significant effects in men.

These findings may reflect differences in the effectiveness of harm reduction strategies depending on the context of drinking. Some level of alcohol use is normative among U.S. college students (Patrick et al., 2024) and is often linked to increased peer connectedness (Paige et al., 2025). Moreover, social motives are the most endorsed type of drinking motive among college students (Van Damme et al., 2013). Women with low social drinking motives who still drink frequently may be engaged in alcohol use outside of typical social contexts (such as drinking alone), where harm reduction strategies may be less effective or harder to implement (e.g., monitoring one’s drink is irrelevant in solo drinking situations).

In contrast, women with strong social drinking motives who also employ harm reduction strategies may be more successful at reducing their drinking than women with low social motives because of an effective reliance on their social circle for support. For example, strategies such as ensuring one goes home with a friend may be more impactful among those who drink within a social group, potentially facilitating earlier departures from drinking contexts and ultimately reducing overall alcohol consumption compared to women who do not have high social motives to drink. These nuanced interactions suggest that both the context and motives for drinking play critical roles in moderating the impact of harm reduction strategies on alcohol use among women.

### Drinking days: Greater other substance use and stronger enhancement motives are associated with more drinking days

For the number of drinking days, there was a main effect of other substance use, such that emerging adults who endorsed more substance use (e.g., cannabis, nicotine, and illicit drug use), also reported a higher number of drinking days, on average. This corroborates a large literature documenting positive associations between recent alcohol and other substance use (O’Hara et al., 2016; Stevens et al., 2022), especially during emerging adulthood. Additionally, enhancement motives were positively associated with the number of drinking days, consistent with findings from a meta-analysis comparing several types of drinking motives where enhancement motives in particular were the strongest predictor of alcohol use (Bresin and Mekawi, 2021). Taken together, our findings suggest that emerging adults who endorse past month use of cannabis, nicotine, and/or other illicit drug use and/or who report higher drinking enhancement motives are also likely to report more frequent alcohol use.

### Drinks per drinking day: Association between limiting/stopping strategies and drinks per drinking day depends on social motives and on sex

When predicting drinks per drinking day, significant 2-way interactions were found between limiting/stopping strategies and social motives, as well as between limiting/stopping strategies and sex. Specifically, among individuals with lower social motives, greater use of limiting/stopping strategies was associated with more drinks per drinking day. In contrast, among those with greater social drinking motives, greater use of limiting/stopping strategies was associated with fewer drinks per drinking day. This is similar to our results for drinks, further suggesting that protective behavioral strategies are most effective when aligned with strong social motives for drinking. Most young adults who drink only do so in social settings (Creswell, 2021), whereas drinking alone is relatively infrequent and has been strongly associated with a wide range of adverse outcomes, including heavy drinking and alcohol problems (Corbin et al., 2020; King et al., 2026). Our pattern of results may be reflective of typical versus atypical/unhealthy drinking patterns. Young adults who report greater social drinking motives may have a more *proactive* approach to drinking, anticipating social drinking occasions with high potential for heavy drinking, in turn planning ahead to engage in limiting/stopping strategies. Prior work suggests that individuals often increase their use of protective strategies in preparation for social events such as 21st birthdays and spring break (e.g., Lewis et al., 2015). In contrast, individuals who report low social drinking motives may be less able to anticipate specific drinking occasions that carry risk for heavy drinking. Thus, when they attempt to engage in limiting/stopping drinking, they are doing so in a *reactive* manner: they have already consumed a high number of drinks. This interpretation is consistent with previous research that has reported strong positive links between reactive sexual assault protective behavioral strategies and sexual assault outcomes (Shaw et al., 2023). Research that uses methods such as ecological momentary assessment to disentangle drinking contexts and acute risk processes are needed to shed more light on these processes.

We also found a moderating effect of sex. However, the direction of this effect was somewhat unexpected, though we note that when examining men and women separately, associations between limiting/stopping motives and drinks per drinking day were not significant. At trend-level, in women, greater use of limiting/stopping strategies was associated with more drinks per drinking day. Though just a trend, this is consistent with prior research suggesting that drinking contexts are complex (Creswell, 2021), and many factors may influence the effectiveness of protective behavioral strategy use. For instance, women may resort to limiting/stopping strategies after drinking has escalated, rendering this strategy ineffective, in line with previous work that has found links between endorsement of protective behavioral strategies and increased risk outcomes (Shaw et al., 2023).

### Alcohol-related consequences: Association between conformity motives and alcohol-related consequences varies for men and women at different levels of limiting/stopping strategies

In examining alcohol-related consequences, there was a significant 3-way interaction with limiting/stopping strategies, conformity motives, and sex, meaning that the interplay between protective behavioral strategies and drinking motives differs somewhat between men and women. For women, greater conformity motives were associated with greater consequences at low and average levels of limiting/stopping strategies. For men, greater conformity motives were associated with greater consequences at average and high levels of limiting/stopping strategies.

Previous research has shown mixed results regarding sex as a moderator in the relationship between protective behavioral strategies and alcohol-related consequences (Benton et al., 2004; Lewis et al., 2010; Sutfin et al., 2009). The present study expands on this literature by emphasizing the importance of drinking motives—specifically conformity motives—in shaping sex-specific patterns of risk and protection. College is an especially appropriate context in which to examine these constructs (conformity motives in particular) because alcohol use is ubiquitous and is attributable, in part, to frequent opportunities to consume alcohol, particularly during social events (Chan et al., 2023; White et al., 2006). Because of this, students might believe that drinking is normal and expected. They also face higher social pressures, such as fitting in with their peers. Such factors can influence their drinking behaviors as they explore and develop their adult identities (Borsari et al., 2007).

Among women, the effect of conformity motives on alcohol-related consequences was apparent when women reported average to low levels of limiting/stopping strategies. This suggests that women who are motivated to conform to social drinking norms but do not frequently engage in protective strategies are especially vulnerable to negative alcohol-related outcomes. In contrast, when women reported high use of limiting/stopping strategies, conformity motives no longer predicted alcohol-related consequences, indicating that these protective strategies may buffer the impact of social pressure to drink. This supports a literature that has documented a consistent, direct link between endorsement of alcohol protective behavioral strategies and reductions in alcohol-related consequences (Pearson, 2013; Peterson et al., 2021). Additionally, our work supports a previous moderation model that demonstrated that individual differences in facets of self-control may help mitigate negative consequences associated with emerging adult drinking (Paige et al., 2023).

For men, the relationship between conformity motives and alcohol-related consequences strengthened with greater use of limiting/stopping strategies. On the one hand, this is inconsistent with past literature that has reported protective effects of alcohol protective behavioral strategies on adverse consequences associated with drinking (Pearson, 2013). On the other hand, some previous work has found links between endorsement of protective behavioral strategies and increased risk outcomes (Shaw et al., 2023). A possible explanation for our positive association at average to high levels of limiting/stopping strategies is that men may employ limiting/stopping protective behavioral strategies only after their drinking has escalated to a hazardous level, at which point it may be too late. Additionally, men may not be implementing these strategies with sufficient skill so as to effectively mitigate risk. For example, having a friend let you know you have had enough to drink may not result in lowered risk when you are surrounded by friends who are also drinking heavily and you are motivated to socially conform. Again, it is important to highlight that these social risk scenarios are complex (Creswell, 2021) and many factors may influence the success of protective behavioral strategies.

### Strengths and limitations

This study had several strengths. First, the explicit focus on sex differences allowed for direct testing between men and women. Second, our inclusion criteria required hazardous levels of alcohol use, ensuring that findings are more directly applicable to individuals who are demonstrating hazardous drinking. Third, we utilized *k*-fold cross-validation, which added a layer of methodological rigor to the study by enhancing the robustness and reliability of results through mitigation of overfitting. Thus, our study’s conclusions are more likely to be replicable. Fourth, the examination of multiple types of alcohol-related outcomes provides a more comprehensive and nuanced understanding of drinking behaviors. Additionally, protective behavioral strategies are important to examine in the context of drinking motives because they have the potential to override the impact of motivations for hazardous drinking, or conversely, strong drinking motives could override the positive effects of protective strategies. This multifaceted approach helps to understand exactly why college students are drinking and what outcomes different motives and protective behavioral strategies lead to, and how these differ between men and women.

There are a few limitations of this study to keep in mind. First, given the relatively small sample size and since all participants were college students who endorsed hazardous levels of alcohol use, results may not generalize well to young adults who are not in college or college students who do not endorse hazardous drinking. Indeed, the college environment presents situations that are not necessarily accessed by non-college individuals (e.g., fraternity and sorority parties) or college students who do not drink to problematic levels. Further, participants were from a single large, public university in the Midwest, and the drinking culture at a particular campus may vary by regionality and student body (Tyler et al., 2017). Second, our cross-sectional design does not permit inferences about causality, and so we do not know whether endorsing drinking motives and engaging in protective behavioral strategies caused the various drinking outcomes. Third, participants reported on past 30-day beliefs and activities, which is a relatively short time frame that may be overly dependent on situational factors such as what time of year it was and what else happened at the time (e.g., final exams, football season). Finally, as is common in this area of research, participants self-reported their drinking behaviors, which could be biased due to social desirability. However, participants recorded their responses directly into online questionnaires, thereby limiting this effect.

## Conclusions

This study expands on previous research by demonstrating that protective behavioral strategies operate within broader motivational and social contexts, such that their associations with alcohol outcomes depend on drinking motives and sex, thereby contributing to a more nuanced understanding of how these factors interact in a complex manner. Strategies to limit or stop drinking or reduce harm from drinking were differentially associated with alcohol outcomes based on individuals’ reasons for drinking. This pattern is consistent with conclusions from reviews by Cox et al. (2024) and Peterson et al. (2021), which emphasize that protective behavioral strategies are best understood as context-dependent behaviors rather than static protective traits. These reviews highlight that some strategies, particularly harm reduction and limiting/stopping, may be used reactively, co-occur with heavier drinking, or be less effective in high-risk or socially pressured situations, even while protective overall. Together, these results underscore the importance of tailoring intervention approaches to individuals’ motivations and drinking contexts. Future research that more closely examines complexities such as drinking context (e.g., bars, parties, home alone), social structures (e.g., sororities/fraternities, dorm living), mental health comorbidities, and physiological responses to alcohol is needed. In addition, future research that uses methods such as ecological momentary assessment to disentangle acute risk processes and efforts at reducing drinking are needed to shed more light on these questions among hazardous college drinkers, as it could help clarify when and for whom protective behavioral strategies are being used effectively.

## Supplementary Material

Supp 1

Supplemental data for this article can be accessed online at https://doi.org/10.1080/10826084.2026.2682302.

## Figures and Tables

**Table 1.** Participant demographics.

	Total sample (*N* = 109)	Women (*n* = 56)	Men (*n* = 53)

**Age (years)**	19.4 ± 0.8	19.5 ± 0.9	19.4 ± 0.8
**Race/Ethnicity**			
White	74	37	37
Asian	18	10	8
Two or more	6	2	4
Latino/a	6	4	2
Black	2	1	1
Other	2	1	1
Pacific islander	1	1	0
**AUDIT-C** *	6.99 ± 1.58	6.54 ± 1.49	7.47 ± 1.55
**Substance Use Composite**	0.00 ± 0.64	−0.17 ± 0.40	0.18 ± 0.78

Numbers for age and the substance use composite (see Other Substance Use in Materials and Methods for details) are means with standard deviations following ±.

Numbers for race/ethnicity are counts.

* Collected at screening.

**Table 2.** Zero-order correlations among study variables.

	1	2	3	4	5	6	7	8	9	10	11

1. Substance Use Composite											
2. Drinks	0.344***										
3. Drinking Days	0.310**	0.692***									
4. Drinks per Drinking Day	0.192*	0.540***	−0.044								
5. RAPI	0.240*	0.226*	0.155	0.161							
6. DMQ Conformity	0.09	0.044	0.042	0.028	0.400***						
7. DMQ Coping	0.174	0.166	0.195*	0.021	0.373***	0.476***					
8. DMQ Enhancement	0.244*	0.343***	0.250**	0.158	0.203*	0.149	0.336***				
9. DMQ Social	0.132	0.342***	0.216*	0.214*	0.233*	0.351***	0.452***	0.549***			
10. PBSS Harm Reduction	−0.111	−0.052	−0.082	0.053	−0.036	−0.14	−0.174	−0.078	−0.035		
11. PBSS Limiting/Stopping	0	−0.222*	−0.153	−0.032	−0.085	0.004	−0.175	−0.099	−0.212*	0.322***	
12. PBSS Manner of Drinking	−0.278**	−0.324***	−0.221*	−0.139	−0.031	−0.061	−0.250**	−0.403***	−0.270**	0.292**	0.375***

*** *p* < 0.001.

** *p* < 0.01.

* *p* < 0.05.

DMQ = Drinking Motives Questionnaire; PBSS = Protective Behavioral Strategies Scale; RAPI = Rutgers Alcohol Problems Index.

**Table 3.** Sex comparisons on variables of interest.

				Welch’s *t*-test		
	Total sample (*N* = 109)	Women (*n* = 56)	Men (*n* = 53)	*t* (*df*)	*p*-value	*d*

**Alcohol Outcomes**						
Drinks	29.14 ± 20.58	23.75 ± 18.32	34.83 ± 21.46	−2.89 (102.41)	**.005**	.56
Drinking Days	6.72 ± 3.66	5.82 ± 3.25	7.68 ± 3.86	−2.71 (101.87)	**.008**	.52
Drinks per Drinking Day	4.38 ± 2.18	3.94 ± 2.16	4.86 ± 2.11	−2.24 (106.89)	**.027**	.43
RAPI	4.52 ± 4.58	4.52 ± 5.16	4.53 ± 3.93	−0.01 (102.43)	.991	<.01
**PBSS Subscales**						
Harm Reduction	8.65 ± 1.73	9.11 ± 1.29	8.17 ± 2.00	2.89 (88.12)	**.005**	.56
Limiting/Stopping	17.83 ± 4.78	18.61 ± 4.25	17.02 ± 5.19	1.74 (100.54)	.085	.33
**DMQ Subscales**						
Conformity	7.51 ± 2.82	7.34 ± 2.53	7.70 ± 3.10	−0.66 (100.44)	.511	.13
Coping	9.38 ± 3.74	9.48 ± 3.96	9.26 ± 3.52	0.30 (106.58)	.762	.06
Enhancement	15.91 ± 3.97	15.16 ± 3.92	16.70 ± 3.90	−2.05 (106.72)	**.043**	.39
Social	18.58 ± 3.88	18.45 ± 4.16	18.72 ± 3.60	−0.36 (106.17)	.717	.07

Numbers are means with standard deviations following ±. *d* = Cohen’s *d*; *df* = degrees of freedom; DMQ = Drinking Motives Questionnaire; PBSS = Protective Behavioral Strategies Scale; RAPI = Rutgers Alcohol Problems Index. Bold values indicate statistical significance at the p < .05 level.

**Table 4.** Model statistics (with covariates).

	Drinks			Drinking Days			Drinks per Drinking Day			RAPI (controlling for drinks)		
*Predictors*	*Estimates (95% CI)*	*Statistic*	*p*	*Estimates (95% CI)*	*Statistic*	*p*	*Estimates (95% CI)*	*Statistic*	*p*	*Estimates (95% CI)*	*Statistic*	*p*

Intercept	3.14 (2.95–3.34)	31.63	**<.001**	1.95 (1.84–2.07)	32.90	**<.001**	3.66 (3.06–4.25)	12.14	**<.001**	3.20 (1.66–4.74)	4.13	**<.001**
Group	−0.11 (−0.35–0.13)	−0.92	.356	−0.13 (−0.34–0.07)	−0.131	.190	0.48 (−0.31–1.27)	1.20	.232	2.60 (0.64–4.56)	2.63	**.001**
Substance Use	0.21 (0.03–0.39)	2.25	**.024**	0.17 (0.03–0.32)	2.35	**.019**	0.24 (−0.38–0.85	.77	.443	1.56 (0.22–2.89)	2.31	**.023**
Drinks										0.04 (−0.01–0.09)	1.59	.116
Sex (Men)	0.37 (0.13–0.61)	3.00	**.003**				0.67 (−0.10–1.45)	1.73	.087	−1.55 (−3.41–0.32)	−1.64	.103
DMQ Conformity										2.41 (0.11–4.70)	2.08	**.040**
DMQ Enhancement				0.10 (0.00–0.21)	1.97	.049						
DMQ Social	0.36 (0.18–0.54)	3.88	**<.001**				0.36 (−0.02–0.74)	1.89	.062			
PBSS Harm Reduction	0.12 (−0.11–0.35)	1.06	.288									
PBSS Limiting/Stopping							0.50 (−0.10–1.09)	1.66	.100	−1.48 (−2.88– −0.08)	−2.09	**.039**
DMQ Conformity × Sex										−0.27 (−2.64–2.11)	−0.22	.824
DMQ Social × Sex	−0.12 (−0.37–0.14)	−0.90	.366									
DMQ Social × PBSS Harm Reduction	−0.45 (−0.67– −0.23)	−4.03	**<.001**									
DMQ Social × PBSS Limiting/Stopping							−0.72 (−1.09– −0.34)	−3.77	**<.001**			
PBSS Harm Reduction × Sex	−0.05 (−0.32–0.22)	−0.35	.725									
PBSS Limiting/Stopping × Sex							−0.89 (−1.67– −0.12)	−2.30	**.023**	1.17 (−0.43–2.76)	1.45	.149
DMQ Conformity × PBSS Limiting/Stopping										−1.87 (−3.97–0.22)	−1.77	.080
DMQ Conformity × PBSS Limiting/Stopping × Sex										2.96 (0.63–5.28	2.52	**.013**
DMQ Social × PBSS Harm Reduction × Sex	0.43 (0.16–0.70)	3.13	**.002**									
R^2^ / R^2^ adjusted	.992 / .992			.638 / .624			.277 / .226			.411 / .351		

CI = confidence intervals; DMQ = Drinking Motives Questionnaire; PBSS = Protective Behavioral Strategies Scale; RAPI = Rutgers Alcohol Problems Index.
