# Supplementary material for: Sex Differences in the Association Between Drinking Motives, Protective Behavioral Strategies, and Alcohol Outcomes in a Hazardous Drinking College Sample
Source: Subst Use Misuse. Author manuscript; Available in PMC 2026 Jul 22. (PMC13389929; doi:10.1080/10826084.2026.2682302)
Supplement: Supp 1 [file NIHMS2189867-supplement-Supp_1.docx]

**SUPPLEMENTAL MATERIAL**

**METHODS AND MATERIALS**

109 emerging adults between the ages of 18 and 20 years were recruited from a single, large midwest university. College-aged emerging adults aged 21 years and over were excluded because reaching the legal drinking age is a significant developmental milestone that can change the social context of alcohol use. At ages 21–22, there is a general stabilization of drinking frequency but a decline in quantity per occasion. The prevalence of binge drinking also tends to peak around age 21 and then begins to decline (e.g., Fromme et al., 2010). Including individuals 21 years and older could potentially confound study results by changing the dynamics of alcohol consumption. Because the broader study was primarily a neuroimaging study, significant changes in alcohol consumption patterns could affect brain structure and function.

40 participants (21 women) were recruited from the Brief Alcohol Screening and Intervention in College Students (BASICS) program offered through the university’s wellness services (intervention group). This group comprised students who were adjudicated into a university-sanctioned brief intervention for hazardous drinking and agreed to participate in the research study. Our study team received a list weekly of students who would be entering BASICS within the next 3–5 weeks. We then sent these students an email with information about our research study and a link to our online screening survey (Qualtrics). BASICS was not delivered nor controlled by our study, and the decision to participate or not participate in the research study did not impact students’ enrollment in the BASICS program. Members of the control group (*n* = 69; 35 women) were recruited through a university research study website and campus flyers. Flyers directed potential control group participants to the same online screening survey mentioned above to determine eligibility, and we contacted those who met inclusion criteria. Members of the control group also met criteria for hazardous drinking but had not been adjudicated to any intervention programs. For both groups, after eligibility was determined via the online screening survey, study team members called potential participants to confirm eligibility and schedule their first visit (Time 1). Inclusion and exclusion criteria are listed below.

The analyses presented here are only from Time 1 data, prior to any intervention for the intervention group. The broader study comprised four total sessions, with each time point coming approximately 4 weeks after the previous one. At the first two time points, participants underwent functional magnetic resonance imaging and filled out measures pertaining to alcohol and other substance use, alcohol-related problems, drinking motives, cravings, and social/emotional functioning. At Time 3 and Time 4, measures pertaining to alcohol and other substance use, alcohol-related problems, drinking motives, cravings, and social/emotional functioning were again collected. The intervention group participated in the BASICS brief intervention program between Times 1 and 2. The control group received a digital pamphlet encouraging lower-risk drinking between Times 1 and 2. This digital pamphlet was the same as what the intervention group received after their first BASICS visit (which occurred after Time 1 for the current study).

Time 1 data were collected on 110 participants; however one participant withdrew during the session before data collection was complete (control group, woman). For the variables included in analyses (PBSS, DMQ), there are no missing data. All data were collected in-person in one session, with the exception of AUDIT-C, which was collected via the screening survey.

*Inclusion criteria:* Both groups had to meet the following inclusion criteria: 1) screen positive for hazardous drinking via the Alcohol Use Disorders Identification Test–Consumption (AUDIT-C; Bush et al., 1998; women had to score ≥4 and men had to score ≥5), 2) be enrolled full-time in college, and 3) be 18–20 years old. *Exclusion criteria:* 1) neurological, acute, uncorrected, or chronic medical illness, 2) current treatment for or diagnosis of any of the following: schizophrenia, post-traumatic stress disorder, bipolar disorder, major depressive disorder, an eating disorder, obsessive compulsive disorder, panic disorder, social phobia, agoraphobia, or generalized anxiety disorder, 3) turning 21 years of age less than 4 months before Time 1; 4) magnetic resonance imaging contraindications such as metal implants, medical devices, pregnancy, previous abnormal MRI, or claustrophobia, and 4) left-handedness or ambidexterity. The latter two criteria are due to the broader study protocol also including neuroimaging.

When examining for group differences on primary study variables (i.e., drinking motives, PBSS, and the proportion of male participants and female participants in each group), there was only a significant group difference for PBSS Limiting/Stopping Drinking (*t*(97.3) = -3.05, *p* = .002), where the intervention group had higher scores than the control group.

**RESULTS**

**Regression Models: Group & Other Substance Use Not Included**

See Supplementary Table 1 for model statistics.

*Drinks*

     The best-fitting model predicting drinks (*R*^2^_adj_ = .990) included a significant 3-way interaction between DMQ Social x PBSS Harm Reduction x sex (*p* = .001). There was also a significant 2-way interaction between DMQ Social x PBSS Harm Reduction (*p* < .001). There were also significant main effects of sex (*p* < .001) and DMQ Social (*p* < .001). Although significant heteroskedasticity was observed in our best fitting model (*p* = 0.24), the usage of a zero-inflated negative binomial model prevented our ability to implement the correction used with the other models, and as a result is presented uncorrected.

*Drinking Days*

     The best-fitting model predicting drinking days (*R*^2^_adj_ = .577) included a significant main effect of DMQ Enhancement (*p* = .018).

*Drinks per Drinking Day*

     The best-fitting model predicting drinks per drinking day (*R*^2^_adj_ = .227) included two significant 2-way interactions: DMQ Social x PBSS Limiting/Stopping (*p* < .001) and PBSS Limiting/Stopping x sex (*p* = .031). There was also a significant main effect of sex (*p* = .044).

*Alcohol-related Problems*

The best-fitting model for RAPI total score (*R*^2^_adj_ = .214) included a significant 3-way interaction of DMQ Conformity x PBSS Limiting/Stopping x sex (*p* = .024) and a significant main effect for DMQ Conformity (*p* = .029). All values reported for alcohol-related problems were corrected using robust estimation to account for heteroskedastic error.

**Supplementary Table 1.** Model statistics (without covariates)

|  | **Drinks** | | | **Drinking Days** | | | **Drinks per Drinking Day** | | | **RAPI** | | |
| --- | --- | --- | --- | --- | --- | --- | --- | --- | --- | --- | --- | --- |
| *Predictors* | *Estimates*  *(95% CI)* | *Statistic* | *p* | *Estimates*  *(95% CI)* | *Statistic* | *p* | *Estimates*  *(95% CI)* | *Statistic* | *p* | *Estimates*  *(95% CI)* | *Statistic* | *p* |
| Intercept | 3.07  (2.89–3.25) | 34.04 | **<.001** | 1.81 (1.67–1.96) | 24.38 | **<.001** | 3.79 (3.27–4.30) | 14.53 | **<.001** | 4.89 (3.43–6.34) | 6.66 | **<.001** |
| Sex (Male) | 0.45  (0.21–0.61) | 3.70 | **<.001** | 0.20  (-0.00–0.40) | 1.93 | .053 | 0.77  (0.02–1.51) | 2.04 | **.044** | -0.50  (-2.25–1.25) | -0.57 | .572 |
| DMQ Conformity |  |  |  |  |  |  |  |  |  | 2.76  (0.28–5.23) | 2.21 | **.029** |
| DMQ Enhancement |  |  |  | 0.13  (0.02–0.23) | 2.37 | **.018** |  |  |  |  |  |  |
| DMQ Social | 0.38  (0.20–0.57) | 4.11 | **<.001** |  |  |  | 0.37  (-0.00–0.75) | 1.96 | .053 |  |  |  |
| PBSS Harm Reduction | 0.13  (-0.11–0.36) | 1.04 | .300 |  |  |  |  |  |  |  |  |  |
| PBSS Limiting/ Stopping |  |  |  |  |  |  | 0.54  (-0.05–1.13) | 1.81 | .073 | -1.23  (-2.68– 0.22) | -1.68 | .096 |
| DMQ Conformity x PBSS Limiting/ Stopping |  |  |  |  |  |  |  |  |  | -1.98  (-4.05–0.08) | -1.91 | .060 |
| DMQ Conformity x Sex |  |  |  |  |  |  |  |  |  | -0.92  (-3.55–1.72) | -0.69 | .493 |
| DMQ Social x PBSS Harm Reduction | -0.44  (-0.66– -0.22) | -3.85 | **<.001** |  |  |  |  |  |  |  |  |  |
| DMQ Social x PBS Limiting/Stopping |  |  |  |  |  |  | -0.74  (-1.11– -0.37) | -3.95 | **<.001** |  |  |  |
| DMQ Social x Sex | -0.10  (-0.36–0.15) | -0.79 | .429 |  |  |  |  |  |  |  |  |  |
| PBSS Harm Reduction x Sex | -0.07  (-0.34–0.21) | -0.49 | .623 |  |  |  |  |  |  |  |  |  |
| PBSS Limiting/ Stopping x Sex |  |  |  |  |  |  | -0.85  (-1.61– -0.08) | -2.19 | **.031** | 1.15  (-0.57–2.86) | 1.33 | .188 |
| DMQ Conformity x PBSS Limiting / Stopping x Sex |  |  |  |  |  |  |  |  |  | 2.90  (0.40–5.40) | 2.30 | **.024** |
| DMQ Social x PBSS Harm Reduction x Sex | 0.44  (0.17–0.72) | 3.13 | **.002** |  |  |  |  |  |  |  |  |  |
| R^2^ / R^2^ adjusted | .991 / .990 | | | .589 / .577 | | | .263 / .227 | | | .265 / .214 | | |

CI = confidence intervals; DMQ = Drinking Motives Questionnaire; PBSS = Protective Behavioral Strategies Scale; RAPI = Rutgers Alcohol Problems Index

**SUPPLEMENTARY REFERENCES**

Bush, K., Kivlahan, D.R., McDonell, M.B., Fihn, S.D., Bradley, K.A., 1998 The AUDIT alcohol consumption questions (AUDIT-C): an effective brief screening test for problem drinking. Ambulatory Care Quality Improvement Project (ACQUIP). Alcohol Use Disorders Identification Test. Arch Intern Med. 58(16), 1789–95. doi: 10.1001/archinte.158.16.1789.

Fromme, K., Wetherill, R.R., Neal, D.J., 2010. Turning 21 and the associated changes in drinking and driving after drinking among college students. J Am Coll Health. 59(1), 21–7. doi: 10.1080/07448481.2010.483706.
